# “Bisphenol a: an emerging threat to male fertility”

**DOI:** 10.1186/s12958-018-0447-6

**Published:** 2019-01-20

**Authors:** Federica Cariati, Nadja D’Uonno, Francesca Borrillo, Stefania Iervolino, Giacomo Galdiero, Rossella Tomaiuolo

**Affiliations:** 1 Dipartimento di Medicina Molecolare e Biotecnologie Mediche, Università degli Studi di Napoli Federico II, Via Sergio Pansini, 5 –, 80131 Naples, Italy; 2 CEINGE-Biotecnologie Avanzate s.c.a r.l., Naples, Italy; 3 KronosDNA s.r.l., Spin-off Università degli Studi di Napoli Federico II, Naples, Italy; 4 Dipartimento di Medicina Clinica e Chirurgia, Università degli Studi di Napoli Federico II, Naples, Italy

**Keywords:** Bisphenol a, Male fertility, Endocrine disrupting compounds

## Abstract

**Background:**

Among the factors causing male infertility, one of the most debated is the exposure to environmental contaminants. Recently, the chemical compound Bisphenol A (BPA) has drawn attention from the reproductive science community, due to its ubiquitous presence in day-to-day life. Its toxic action appears to mainly affect the male reproductive system, directly impacting male fertility.

**Main:**

The purpose of this review is to investigate current research data on BPA, providing an overview of the findings obtained from studies in animal and human models, as well as on its supposed mechanisms of action.

**Conclusion:**

A clear understanding of BPA action mechanisms, as well as the presumed risks deriving from its exposure, is becoming crucial to preserve male fertility. The development and validation of methodologies to detect BPA toxic effects on reproductive organs can provide greater awareness of the potential threat that this chemical represents.

Bisphenol A (BPA) is used in industry, especially in polycarbonate plastics manufacturing processes and food packaging [1]. BPA is a crystalline chemical compound with formula C_15_H_16_O_2_ and a structure made of two hydroxyphenyl groups, which give to it a mild phenolic odour. BPA-based polycarbonate plastics are exceptionally strong and stable as they can endure exposure to high temperatures and sustain high-impact collisions. These characteristics make them valuable as components of safety equipment and food containers as they withstand heating in microwave ovens. Being a component of epoxy resins in protective coatings, such as those lining the inner surfaces of cans, BPA helps to extend the shelf life of food and beverage products. Indeed, one of the first studies aimed at quantifying BPA leaching from food containers showed that the chemical is present at a range of 4-23 μg per can [2]. The resiliency of BPA plastics has led to their use in medical devices such as heart-lung machines, incubators, hemodialyzers, and dental sealants and fillers; also, their light weight and optical clarity has made them especially useful for eyeglasses. Furthermore, BPA is found in a variety of other products, including compact discs and paper receipts.

Due to his widespread applications, the use of BPA has garnered increasing attention over the last decade, especially in terms of human safety. It has been estimated that levels of conjugates of BPA in urine are above safety thresholds in 90% of individuals tested in several population studies [3].

Unconjugated BPA, in its aglycone form, was shown to bind estrogen receptors, resulting in a weak estrogenic activity [4, 5]. At the same time, experimental studies in animal models reported BPA ability to bind to androgen receptors (AR), along with other metabolic regulators as thyroid hormone receptors [6]. Due to these results, more attention has been recently focused on BPA toxic effects on the reproductive system [7]. Although scientific data obtained from wildlife and in vivo studies in animal models show the negative effects of BPA on reproductive fitness, there is a growing body of literature investigating the disrupting effects of BPA on male reproductive system, which however presents heterogeneous and sometimes conflicting results between animal and human. This review intends to gather scientific data about the BPA effects on the male reproductive system and the most appropriate analytical strategy. In this review, the effects of BPA on animal and human reproduction and on the hypothalamic-pituitary-gonadal axis will be presented and discussed.

## Mechanisms of BPA as reproductive toxicity

Based on observed evidence from in vitro and in vivo studies, different hypotheses were postulated about the mechanisms through which BPA exerts its toxic effects on reproductive system. In particular, BPA is commonly considered to have estrogenic and antiandrogenic effects able to disrupt the hypothalamic-pituitary-gonadal axis, and the ability to alter normal epigenetic patterns with impairing consequences on the reproductive system.

The processes of gonadotropin-releasing hormone (GnRH) release, gonadotropins secretion, and signalling trigger for spermatogonial cell proliferation in Sertoli cells, can all be affected by BPA action [8]. Gonadotropin levels alteration, in particular a decrease of LH serum concentration, induces a reduction in testosterone production by Leydig cells. Commonly, testosterone is converted to DHT and is responsible for spermatogenesis, transport and storage of spermatozoa prior to ejaculation through the epididymis. Furthermore, testosterone, after its conversion to estradiol by aromatase, maintains Sertoli’s cells functions. Consequently, low testosterone levels and alteration of estradiol catabolism result in high levels of estradiol, which compromise sperm production as demonstrated in young rats treated with high doses of estrogens [9].

It is known that estrogen receptors (ER) are expressed in Leydig cells (ERα), whereas ERβ receptors are expressed in Sertoli cells, pachytene spermatocytes and round spermatidis of the adult rat and male testis. Molecular studies have reported that BPA is a selective ER modulator, which means that it acts as an estrogen agonist in some tissues and an estrogen antagonist in others [10]. In vitro studies have demonstrated that BPA binding to estrogen receptors alters their ability to recruit tissue-specific co-activators important for differential tissue-dependent responses [11, 12]. In addition, it has been demonstrated that BPA has chemical affinity for a membrane-associated G protein-coupled estrogen receptor (GPER), equivalent to its primary ligand, estradiol. By binding to the GPER receptor, which expression has also been identified in the hypothalamus and pituitary, BPA can induce rapid, non-genomic effects [13].

According to previous data, an in vivo study in the adult rat showed that low doses of BPA can induce strong, membrane-initiated estrogenic effects, indicating that the exposure to low levels of this compound might interfere with normal estrogenic signalling pathway [14].

In vivo studies on Wistar rats, performed at different developmental stages, showed that BPA estrogenic effect results in the inhibition of testicular steroidogenesis, leading to hypogonadotropic hypogonadism with development of defective reproductive tracts [15, 16].

Research on BPA antiandrogenic activity has produced controversial data. Chemical substances with antiandrogenic properties are able to modulate male reproductive functions by inhibiting the binding of androgens to AR and subsequently down-regulate androgen-induced gene expression. Most of antiandrogenic chemicals contain at least an aromatic ring with a hydroxyl group (-OH). In the case of BPA, the –OH on the A-phenyl ring is essential for the inhibitory effect on the AR [17]. In vitro studies showed that, following treatment with BPA, the inhibition of AR is partial and it lacks of a dose-response relationship, suggesting a non-competitive mechanism [18]. Contrary, an another in vitro study, showed that BPA is able to block the androgen receptor-mediated gene expression competing with DHT to bind AR, revealing a significant inhibitory effect on the DHT-induced transcriptional activity [19].

Recent data instead showed BPA as an androgen receptor antagonist, preventing endogenous androgens from regulating androgen-dependent transcription and inhibiting Sertoli cell proliferation [20]. The mechanism operates by blocking the amino- and carboxyl-terminal regions (AR N/C) of the AR and enhancing the interactions of AR with silencing mediator for thyroid hormone receptors (SMRT) and nuclear receptor co-repressor (NCoR) [20].

Finally, several in vitro studies suggested that epigenetic alterations might occur following BPA exposure, causing adverse effects of on male reproductive system, including lowering semen quality. Atkinson and colleagues showed that bisphenol o-quinone, a reactive metabolite of BPA, is able to bind DNA by covalent bonds and in the presence of peroxidase activation system also produced toxic adducts [21]. Formation of DNA adducts in sexual tissues throughout organogenesis can cause genetic imbalance, gene modifications and chromosomal mutations with permanent effects [22]. Current studies also suggest that early exposures to BPA could lead to late onset modifications that could be inherited throughout generations by epigenetic mechanisms, such as methylation-meditated promoter silencing [11].

According to in vitro studies, Manikkam and colleagues showed that methylation levels of long interspersed nucleotide elements, which is used as a marker of genome-wide methylation status, is significantly lower in human sperm of BPA-exposed workers compared to unexposed controls [23].

## BPA and male reproduction in animal models

The toxic effect of BPA on the male reproductive functions is well defined in animals model and demonstrated by physiological changes throughout foetal, pubertal and adult life of male rats (Table 1) [15, 24, 25]. In addition several in vitro studies were performed to elucidate the mechanisms through which BPA is able to modify the endocrine response, the effect of steroid hormones as well as spermatogenesis.

**Table 1:**
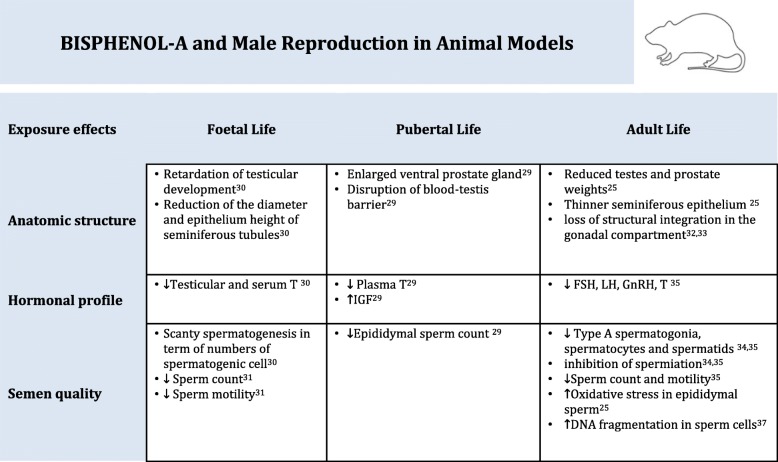
Bisphenol-A and Male Reproduction in Animal Models

↓ decrease, ↑ increase, T Testosterone, IGF Insuline-like Grow Factor, GnRH Gonadotropin Releasing Hormone

It is proven that developing embryos are more vulnerable to environmental contaminants than the adult animals [26]. The extensive evidence, reported above, that BPA exerts estrogenic activity and the possibility that even a low exposure during foetal life could have a toxic effect at several physiological levels is under debate [15].

Several studies confirm that mice treated with BPA, even at low dosage, during the foetal life show persistent effects on tissues of male reproductive organs, structural and neurological changes as well as alteration of androgens functions that play a fundamental role in male sex differentiation and development of the male phenotype [27–29].

In particular, male mice exposed to BPA during the preimplantation period (days 1–5 of gestation), showed a reduction of serum and testicular testosterone levels when euthanized at 24 postnatal days and an increase of GnRH mRNA at 35 and 50 postnatal days [30]. In addition, retardation of testicular development with a reduction of seminiferous tubules’ diameter and epithelium height in BPA-exposed mice (35 postnatal days) and a scanty spermatogenesis in terms of numbers of spermatogenic cells (50 postnatal days) was detected. Finally, a decrease in expression of testicular StAR (responsible for cholesterol transport to the inner mitochondrial membrane), and a reduction of histone acetylation of the StAR gene promoter, was observed in BPA-exposed mice at 35 and 50 postnatal days [30].

Recently, an in vivo study in pregnant mice exposed to BPA on embryonic days 7 to 14 showed testis morphological alteration with a reduction in the number of stage VIII seminiferous epithelial cells and a decrease of sperm count, motility parameters, and intracellular ATP levels in offspring mice analysed at postnatal day 120 [31]. In addition, this study showed a decrease of protein kinase A (PKA) activity and tyrosine phosphorylation in spermatozoa (essential proteins for ATP generation and oxidative stress response).

Contrary, female rats treated with Bisphenol AF (1,1,1,3,3,3-hexafluoro-2,2-bis(4-hydroxyphenyl) propane, BPAF), an analogue of BPA, during gestational and lactation period showed a significant increase of testosterone levels and significant decrease of inhibin B (INHB) levels in offspring’s testis [28]. Moreover, using RNA-seq analysis, BPAF was shown to alter the expression of 279 genes in the testis of pups exposed to BPA both in prenatal and postnatal stages. Especially, expression alteration was detected for those genes involved in G2/M checkpoint, cell differentiation, cell cycle, G2/M transition, and DNA recombination [28]. Specifically, in disaccord with previously mentioned study, these experiments showed that BPAF was able to increase the transcription of StAR, and mRNA levels of ERa and AR. In addition, testes of male rats exposed to BPAF exhibited increased protein levels of genes involved in steroidogenesis (P450scc and StAR) compared to those in the control group [28].

Studies in pubertal male rats showed that exposure to BPA determines an increase of plasma LH after LHRH injection and a reduction of plasma testosterone levels, with a consequent decrease of epididymal sperm count. In addition, an enlarged ventral prostate gland and an increase of plasma IGF-I were observed in BPA-treated rats [29]. The toxic effect of BPA on spermatogenesis is probably due to its ability to perturb the integrity of the blood-testis barrier; in vitro studies on Sertoli cells showed an association between exposure to BPA, ERK pathway activation, a decline in the levels of specific tight junctions proteins, basal ectoplasmic specialization and blood-testis barrier gap junctions [29].

BPA chemical toxic effects are confirmed in adult rats, showing reduced testes and prostate gland weights, decreased serum testosterone levels, reduced diameter and thickness of seminiferous tubules, significantly thinner seminiferous epithelium and subsequent abnormal spermatogenesis in term of decreased sperm count and motility [25]. The authors postulate that in rats exposed to BPA there is a loss of structural integration in the gonadal compartment with the formation of gaps between germinal cells, as demonstrated previously in in vitro studies [32, 33].

Studies focused on the effect of BPA on spermatogenesis revealed a reduction of type A spermatogonia, spermatocytes and spermatids and an inhibition of spermiation, characterized by an increase in stage VII and a decrease in stage VIII of the seminiferous epithelium cycle [34, 35]. In an in vivo study by Jin and colleagues, low dosages of BPA were given to rats via oral administration; results show an impairment of spermatogenesis caused by the reduction of reproductive hormones serum level (FSH, LH, GnRH) and stopping germ cells meiosis process, thus activating the apoptosis pathway in germ cells [35]. In details, BPA administration reduces testosterone biosynthesis and secretion, thus inhibiting the activity of GnRH neurons, and lowering the expression of steroidogenic enzymes. Consequently, a decline of testosterone levels and a reduction in spermatozoa concentration was seen.

Another study, male chicks treated with oral administration of BPA in low doses for more than 23 weeks resulted in developmental arrest and reduced weight of the testes, which showed smaller seminiferous tubules defective spermatogenesis [36].

Additionally, levels of malondialdehyde and superoxide dismutase and decreased levels of glutathione peroxidase were found increased in the liver of BPA-treated rats compared to the control group. This observation leads to the hypothesis that BPA also induces antioxidants depletion and oxidative stress in epididymal sperm [25]. As a result, BPA disrupts the rapid movement of sperm through the epididymis, ultimately compromising its function. Moreover, the oxidative stress caused by BPA alters cellular metabolism, depleting ATP metabolism, affecting the intermediate piece functions and ultimately decreasing spermatozoa motility and velocity [37]. BPA administration in animals was also found associated to significant DNA fragmentation in sperm cells [37]. Additionally, a recent study by D’Cruz et al. suggests that BPA ability of inducing oxidative stress and estrogenic activity may also perturb glucose homeostasis in testes [38].

## BPA and male reproduction in humans

Few studies have investigated BPA exposure in relation to male reproduction in humans, and results are discordant (Table 2).

**Table 2:**
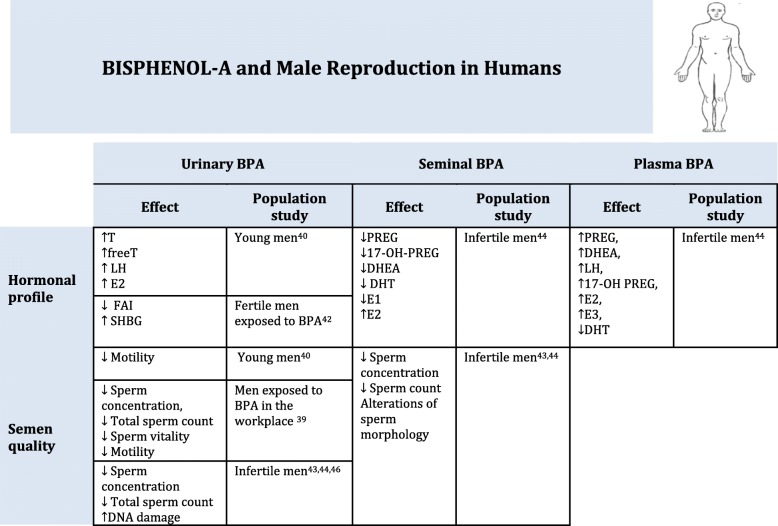
Bisphenol A and Male Reproduction in Humans

↓ decrease, ↑ increase, T Testosterone, LH Luteinizing Hormone, E2 Estradiol, FAI Free Androgen Index, SHBG Sex Hormone Binding Hormone, E1 estrone, E3 estriol, PREG Pregnenolone, DHEA 5-dehydroepiandrosterone, DHT 5a-dihydrotestosterone

An epidemiological study on 218 men showed that men exposed to BPA in the workplace have an increased risk for compromised semen quality compared to men not exposed to BPA. In particular, an increasing urinary BPA level was significantly associated with the decrease of sperm concentration, total sperm count, sperm vitality and motility [39]. In addition, authors showed a dose-response relationship between increasing urine BPA levels and reduction in semen quality in men with low BPA exposure as well as in those with high BPA exposure at the workplace. [39].

Interestingly, Lassen and colleagues, in order to evaluate a possible effect on reproductive hormones as well as semen quality, measured BPA concentration in 308 Danish young men who attended a compulsory physical examination for military service. Authors found urine BPA detectable in 98% of men and an increase of the concentration of serum testosterone, luteinizing hormone (LH), estradiol and free testosterone in BPA dose-dependent manner. In addition, men in the highest quartile of BPA also had significantly lower percentage progressive motile spermatozoa compared with men in the lowest quartile [40]. In contrast with previous study, Lassen at al didn’t found any association between BPA and other semen parameters.

Goldston et al. in a similar population study, 501 males at reproductive age, didn’t find any association between BPA and conventional semen parameters [41]. However, despite the enlarged population study, the semen analysis was performed automatically by CASA system 24 h after collection resulting in a difficult comparison with clinical outcome. Therefore the study is not directly comparable with clinical assessments.

Instead, a study on 375 fertile men, partners of pregnant women, exposed to low environmental BPA levels has shown a significant inverse association between urinary BPA concentration and free androgen index (FAI) levels, as well as a significant positive association between BPA and the sex hormone binding globulin (SHBG) [42]. No significant associations were found between any semen parameters and urinary BPA concentration [42].

On the other hand, BPA in men with impaired fertility appears to alter hormones levels with detriment of semen parameters. Men with different degrees of fertility, classified as slightly (oligospermic, asthenospermic and oligoasthenospermic men), moderately (teratospermic, oligoasthenoteratospermic and oligoteratospermic men) and severely infertile men (azoospermic men), have shown a negative association between seminal BPA levels (but not BPA plasma levels) and sperm concentration, total sperm count and morphology [43, 44]. In addition, hormone measurements have shown a different correlation between plasma and seminal BPA. Specifically, plasma BPA levels were found positively correlated with steroids levels at early stages of hormone biosynthesis (PREG, 17-OH-PREG and DHEA), negatively associated with di 5α-dihydrotestosterone (DHT) and positively associated with estradiol (E2) and estrone (E1). Contrary to plasma associations, seminal BPA levels appear to be negatively associated with steroids levels. Similarly, seminal BPA concentrations were positively correlated with E2 and estriol (E3]. The evident divergence and, sometimes, opposed association between steroids and BPA in both fluids plasma and seminal suggests that their composition is significantly different [44].

Finally, infertile and fertile men from metropolitan, urban and rural Italian areas were enrolled in a study to the investigated BPA levels and expression of nuclear receptors (ERα, ERβ, AR, PXR and AhR). A significant difference was reported in metropolitan areas where infertile men had significantly higher levels of BPA compared to fertile men [45]. In addition, gene expression analysis showed that men from metropolitan areas had higher expression levels of nuclear receptors compared to subjects from other areas. Expression ERα, ERβ, AR, AhR and PXR genes was positively correlated with BPA levels, suggesting their possible use as biomarkers for BPA intoxication.

In addition, an association between BPA urinary concentration and an increase in sperm DNA damage measured as the percentage of DNA in comet tail was found [46]. DNA structure alteration could be attributed to the induction of oxidative stress and depletion of antioxidant defence mechanisms. In an in vitro study by Barbonetti and colleagues, human spermatozoa were exposed to different levels of BPA, starting from concentration of 300 μM. The researchers found that BPA can affect sperm integrity even at low concentrations, due to the formation of pro-oxidants and apoptosis triggered by mitochondrial dysfunction [47]. Exposure to BPA was also associated with an increased mitochondrial generation of superoxide anion, caspase-3 and caspase-9 activation and a sperm motility decline [47]. Importantly, it is well documented that sperm DNA damage is associated to decreased sperm count and increased sperm aneuploidy rates and subsequently associated to atypical telomere lengths (essential for the maintenance of chromosome stability). One of the consequences of paternal genome instability is the disruption of sperm functionality [48]. In support to this hypothesis, clinical data showed that patients with semen samples affected by all of these alterations didn’t produce viable pregnancies. In fact, the demonstrated toxic effect of BPA on semen quality raises the question about potential adverse effects during embryo development. In a study by Knez and colleagues on 149 couples undergoing IVF or intracytoplasmic sperm injection (ICSI) procedures, an association between urinary BPA concentration in the male partner and lower sperm count and concentration was identified. However, no negative effects on embryo development parameters from oocyte fertilization to the blastocyst formation stage were detected [49].

## Discussion

This review focused on BPA toxicity on the reproductive system, focusing on its antiestrogenic and antiandrogenic effects. Especially, experimental evidence and conflicting data about the effect of BPA on male reproduction in animal model and in humans were pointed out. Despite some controversial data, it is clear that BPA-mediated alteration of endogenous steroids levels occurs at different stages as they enter synthesis, metabolism, distribution or clearance processes. BPA can also interact directly with steroid receptors to either simulate or block steroid actions [50, 51]. The majority animal studies, performed at different developmental stages, showed that BPA estrogenic effect results in testis morphological alteration, testicular steroidogenesis inhibition, leading to hypogonadotropic hypogonadism and compromised spermatogenesis. On the other hand, discordant data were reported about the role of BPA in humans. Inconsistency of results regarding BPA effects on semen quality could be due to intrinsic differences in population sampling across the various studies. In fact, all studies reviewed in this article have sampling biases as they analyse men exposed to BPA but with no proven fertility [39], young men not exposed to BPA and with no proven fertility [40] and fertile men from the general population exposed to BPA [42].

In addition, differences in concentrations of individual steroids and BPA across human body fluids were shown by several studies explained above. In particular, concentrations were measured with different methods, introducing potential biases in the analysis and consequently affecting clinical significance. Probably, the development of a method able to measure both steroids and BPA in a single run would deliver more accurate results, as shown for estrogens and BPA by LC–MS/MS assay [52]. In particular, this study found that BPA and steroids concentration differed between seminal fluid and blood plasma. The results of this validation method confirmed that there is a transfer of BPA from blood to seminal plasma through blood–testis barrier, as previously indicated in in vitro studies.

## Conclusion

**Fig. 1:**
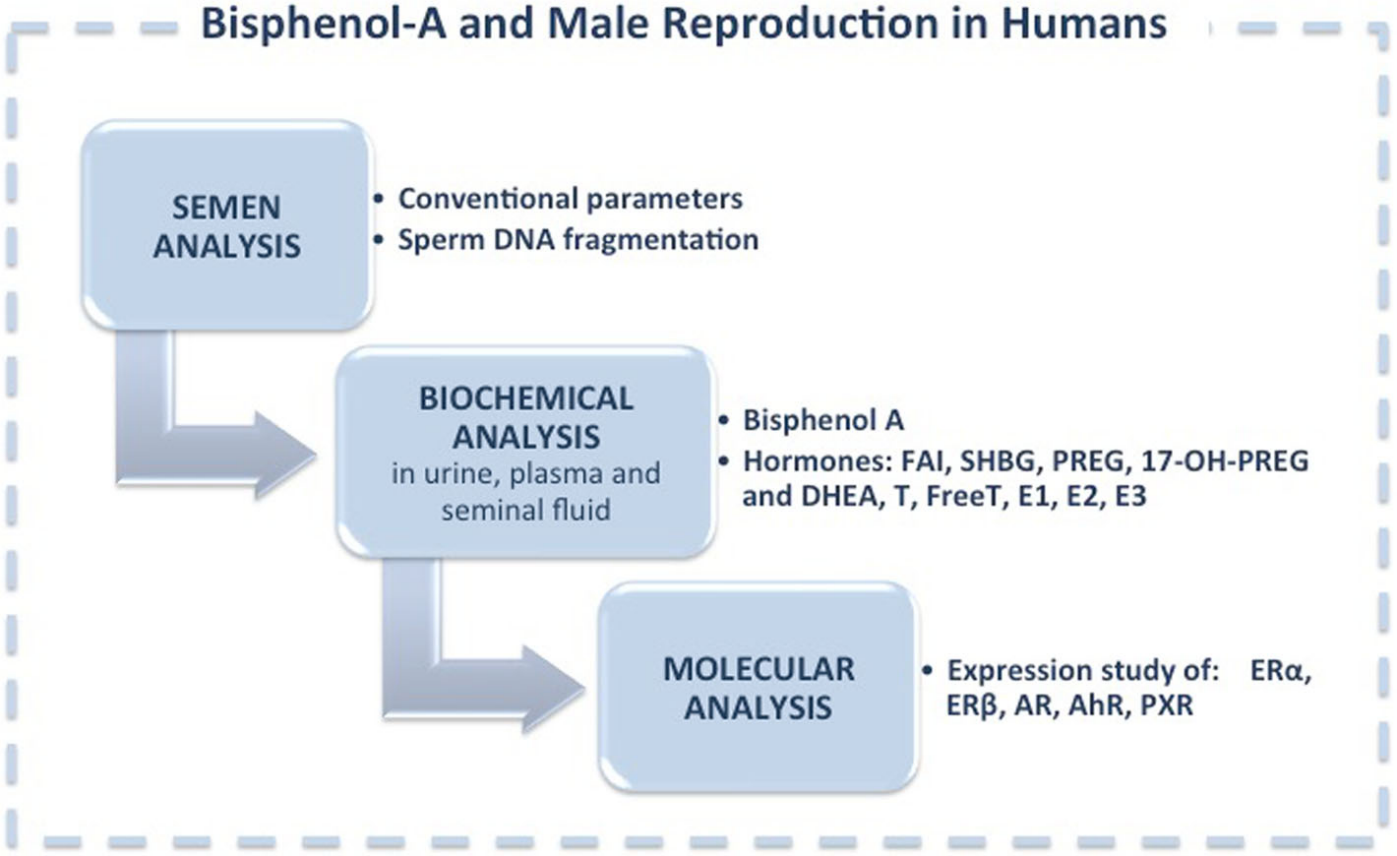
Flowchart of studies on Bisphenol-A and Male Reproduction in Humans

A clear understanding of BPA action mechanisms, as well as of the presumed risks deriving from its exposure, is becoming crucial to preserve male fertility. In order to improve the weight-of-evidence about BPA effects, large population studies aimed to analyse semen parameters, steroid hormone levels and molecular variations of fertile and infertile men is needed as outlined in Fig. 1. In order to properly evaluate BPA toxic effect on reproductive organs, it will be crucial that future studies follow World Health Organization guidelines for semen analysis and employ the most accurate method to measure BPA concentration in seminal fluid.

## Abbreviations

17-OH-PREG: 17α-hydroxypregnenolone
AhR: Aryl hydrocarbon receptor
AR: Androgen receptor
BPA: Bisphenol A
DHEA: 5-dehydroepiandrosterone
DHT: 5α-dihydrotestosterone
EDC: Endocrine disruptors and reproductive health
ER: Estrogen receptor
ERK: Extracellular signal-regulated kinases
FAI: Free androgen index
FSH: Follicle stimulating hormone
GnRH: Gonadotropin-releasing hormone
GPER: Associated G protein-coupled estrogen receptor
ICSI: Intracytoplasmic cell injection
IGF-I: Insulin-like growth factor
INHB: Inhibin B
IVF: In vitro fertilization
LH: Luteinizing hormone
LHRH: Luteinizing hormone releasing hormone
NCoR: Nuclear receptor co-repressor
PREG: Pregnenolone
PXR: Pregnane X receptor
SHBG: Sex hormone binding globulin
SMRT: Silencing mediator for thyroid hormone receptors
